# Conserved Curvature of RNA Polymerase I Core Promoter Beyond rRNA Genes: The Case of the Tritryps

**DOI:** 10.1016/j.gpb.2015.09.005

**Published:** 2015-12-21

**Authors:** Pablo Smircich, María Ana Duhagon, Beatriz Garat

**Affiliations:** 1 Molecular Interactions Laboratory, School of Sciences, University of the Republic, Montevideo 11400, Uruguay; 2 Genetics Departament, School of Medicine, University of the Republic, Montevideo 11800, Uruguay

**Keywords:** Intrinsic curvature, RNA polymerase I, Core promoter, *Trypanosoma*, *Leishmania*

## Abstract

In trypanosomatids, the **RNA polymerase I** (RNAPI)-dependent promoters controlling the ribosomal RNA (rRNA) genes have been well identified. Although the RNAPI transcription machinery recognizes the DNA conformation instead of the DNA sequence of promoters, no conformational study has been reported for these promoters. Here we present the *in silico* analysis of the intrinsic DNA curvature of the rRNA gene **core promoters** in *Trypanosoma brucei*, *Trypanosoma cruzi*, and *Leishmania major*. We found that, in spite of the absence of sequence conservation, these promoters hold conformational properties similar to other eukaryotic rRNA promoters. Our results also indicated that the intrinsic DNA curvature pattern is conserved within the ***Leishmania*** genus and also among strains of *T. cruzi* and *T. brucei*. Furthermore, we analyzed the impact of point mutations on the **intrinsic curvature** and their impact on the promoter activity. Furthermore, we found that the **core promoters** of protein-coding genes transcribed by RNAPI in *T. brucei* show the same conserved conformational characteristics. Overall, our results indicate that DNA **intrinsic curvature** of the rRNA gene **core promoters** is conserved in these ancient eukaryotes and such conserved curvature might be a requirement of RNAPI machinery for transcription of not only rRNA genes but also protein-coding genes.

## Introduction

*Trypanosoma cruzi*, *Trypanosoma brucei,* and *Leishmania major*, which are collectively known as Tritryps, are the etiologic agents of Chagas disease, sleeping sickness, and leishmaniasis, respectively, causing millions of human deaths in tropical and subtropical countries [1]. Trypanosomatids are interesting biological models since they represent a very early branch in the eukaryotic evolution, thus show remarkable deviations from canonical eukaryotic paradigms.

In trypanosomatids, the protein-coding genes are arranged in large directional gene clusters (DGCs) [1]. Despite initiating at still imprecisely defined promoters [2], [3], [4], [5], RNA polymerase II (RNAPII) directs transcription of polycistronic mRNAs in an apparently constitutive manner [6], [7]. Individual mRNAs are later generated by 5′ *trans*-splicing, a process that involves the addition of a small conserved RNA called the spliced leader (SL) and the polyadenylation at their 3′ ends [8]. In this context, regulation of the expression of protein-coding genes in trypanosomatids seems to occur mainly at post-transcriptional levels [6], [7].

As in other eukaryotes, the trypanosomatid RNA polymerase I (RNAPI) drives rRNA gene transcription [9]. The rRNA transcription units are repeated in tandem and separated by intergenic spacer regions. They display a conserved core domain that includes the transcription start point (TSP), as well as upstream and downstream unique and repetitive control elements at variable distances [9]. The nucleotide sequences coding for rRNA genes and the overall organization of their promoters (including *cis*-acting elements and *trans*-acting factors) are evolutionary conserved [10]. However, the elements that mediate transcription initiation at the promoter show no obvious sequence identity across species. Indeed, signals directing protein recognition at the promoter region are constituted by DNA conformations rather than the primary nucleotide sequences [11], [12]. For this reason, the conformational structures of the rRNA gene promoters have been examined in several organisms [11], [12]. As a result, a common structural motif has been described for the rRNA gene conformation, which consists of a flexible structure surrounded by a region with intrinsic curvature at the proximity of TSP. In addition, conserved structural signals have been considered to account for the functional exchangeability of heterologous RNAPI transcription factors in the absence of consensus sequence binding sites [11]. However, no promoter conformational study has been reported for Tritryps so far.

Interestingly, the *T. brucei* RNAPI has the unusual function of directing the transcription of the gene families encoding the developmentally-regulated variant surface glycoproteins (VSGs) and genes encoding the less variable EP/GPEET procyclinss [13]. VSGs are key actors in the evasion of the host immune system, since their protein isoforms are switched at the cell surface during the antigenic variation process. There are 10–20 telomeric bloodstream expression sites (BESs), from which the *VSG* can be transcribed as a polycistron mRNA together with the so-called expression site associated genes (ESAGs), while only one *VSG* variant is expressed at a time [14]. The BES promoters have been found to share a high degree of sequence identity [14], [15]. Upon entering the insect vector, the trypanosomes transform into procyclic forms and, at that point, the gene expression of *VSG*s is silenced and VSGs at the cell surface are replaced by procyclins, another type of stage-specific glycoproteins. These procyclin proteins can be classified into two different types: one containing a domain of tandem pentapeptide repeats (GPEET) and the other characterized by a dipeptide repeat (EP) [13]. Genes encoding procyclins are organized in two distinct loci—*GPEET*/*PAG3* and *EP1*/*PAG1-2*, each harboring a single independent promoter [16]. Afterward, at the salivary gland of the fly vector, the differentiation of procyclic into the metacyclic infective form leads to the expression of a specific set of *VSG* genes, which are distinctively transcribed into monocistronic mRNAs [17]. Although it is estimated that there are 25 metacyclic *VSG* expression sites, sequence data are limited due to the gene assembly difficulties caused by their telomere location [18].

To further the current understanding of the molecular mechanisms involved in transcription initiation by RNAPI in trypanosomatids, we examined whether the core promoters of Tritryp rRNA gene share the conformational characteristics conserved in eukaryotes. To evaluate the conservation of this phenomenon, we extended the analyses to the genus *Leishmania* and also to strains variants of *T. cruzi* and *T. brucei*. Furthermore, the analysis of the previously-reported transcriptionally-deficient single nucleotide mutants at these core promoters showed that changes in curvature produced by base substitutions is sufficient to explain the observed transcriptional failure. Finally, we examined the intrinsic DNA curvature at the core promoter of the protein-coding genes that are distinctively transcribed by RNAPI in *T. brucei*. We found that regardless of the nature of the encoded products (either protein or RNA), the core promoters of the genes transcribed by the trypanosomatid RNAPI are characterized by the presence of the conserved pattern of curvature, as described for other eukaryotes. Altogether, these observations support the importance of promoter curvature for rRNA transcriptional machinery and, for the first time, extend this finding to protein-coding genes.

## Results

### The rRNA gene promoters of *T. cruzi* and *T. brucei* share the eukaryotic curvature

**Supplementary Figure S1:**
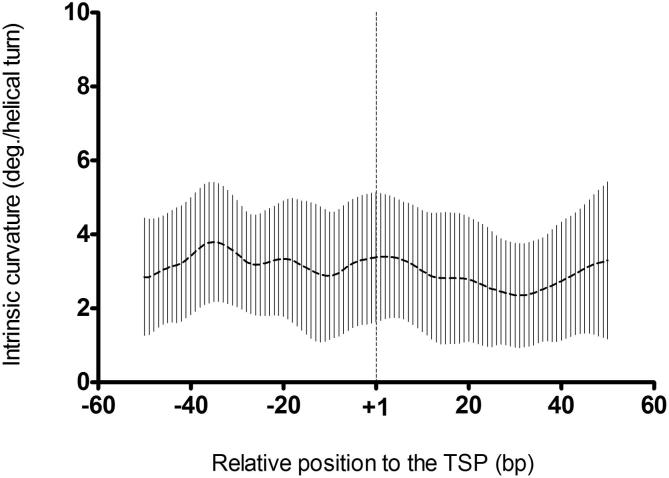
**Average intrinsic curvature for core promoters of human RNAPII genes** The average intrinsic curvature for the core promoters of 35 random RNAPII-transcribed genes in the 100-bp regions centered at the TSP. Dots and vertical bars at each nucleotide position represent average curvature and standard deviation, respectively. See Materials and Methods for database identifiers. The vertical dashed line at +1 marks the position of the TSP. Curvature is expressed as degrees (deg.) per helical turn. TSP, transcription start point.

**Figure 1:**
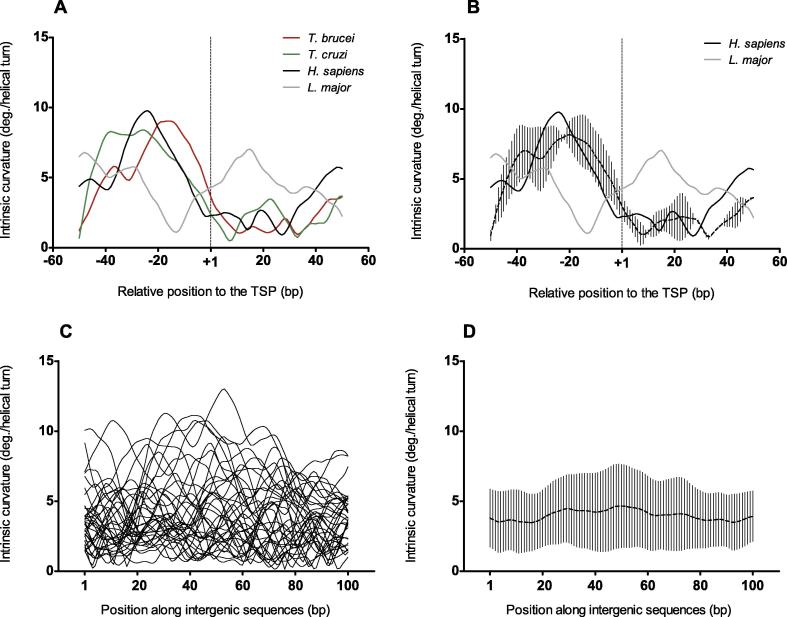
**Intrinsic curvature profiles for rRNA gene promoters in Tritryps** Intrinsic curvature for the core promoters of indicated rRNA genes in the 100-bp regions centered at the TSP (+1) in Tritryps. **A.** Intrinsic curvature of the individual core promoters of rRNA genes from *T. brucei* (red), *T. cruzi* (green), *L. major* (gray), and *H. sapiens* (black). GenBank accession numbers for these sequences are listed in Table 1. **B.** Average intrinsic curvature average of the *T. cruzi* and *T. brucei* core promoters of rRNA genes shown in A. The human profile is shown as a solid black line. The divergent *L. major* profile is shown as a solid gray line. At each nucleotide position, dots and vertical bars represent average curvature and standard deviation respectively. **C.** Intrinsic curvature of 35 randomly-selected intergenic sequences in *T. brucei.***D.** Average intrinsic curvature of the 35 intergenic sequences shown in C*.* In panels B and D, dots and vertical bars at each nucleotide position represent average curvature and standard deviation, respectively. The vertical dashed lines at +1 in A and B mark the position of the TSP. Curvature is expressed as degrees (deg.) per helical turn. TSP, transcription start point.

To start the comparative study of the core promoters of rRNA genes in the Tritryp, we first analyzed their nucleotide sequences. Considering the high intra-species nucleotide conservation among rDNA copies, we selected one representative sequence for each species, whose TSP were experimentally defined, and used the extensively studied human counterpart as an external group. We did not find obvious nucleotide sequence conservation among these rRNA promoters (Table 1). On the contrary, when we compared the intrinsic curvature of the same core promoter sequences, we found that *T. brucei* and *T. cruzi* rRNA promoters showed the conserved eukaryotic DNA structural feature that has been previously described for the corresponding promoters in human [11] (Figure 1**A**). They are characterized by a region of high curvature, peaking consensually at position −20 relative to the TSP, which is followed by a gradual decrease of curvature toward the TSP (Figure 1**B**). Nevertheless, the pattern displayed by *L. major* is dissimilar (Figure 1A), with the minimum curvature value shifted upstream to the TSP. As a control, we analyzed 35 randomly-selected intergenic sequences of trypanosomes. As expected, their curvature consensus was shown to be monotonous (Figure 1**C** and **D**). Similarly, core promoters of 35 randomly-selected eukaryotic sequences transcribed by RNAPII also exhibited a monotonous consensus curvature (Figure S1).

### The curvature of the rRNA gene promoters is conserved within the *Leishmania* genus

**Figure 2:**
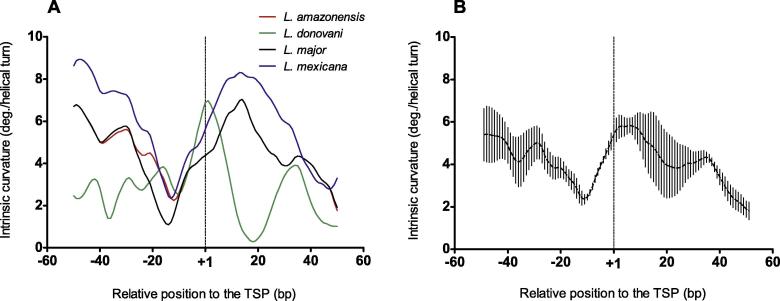
**Intrinsic curvature profiles around the TSPs for rRNA gene promoters in *Leishmania*** Intrinsic curvature for the core promoters of indicated rRNA genes in the 100-bp regions centered at the TSP in *Leishmania*. **A.** Individual intrinsic curvature for the core promoters of selected rRNA genes of several *Leishmania* genera including *T. amazonensis* (red), *L. donovani* (green), *L. major* (black), and *L. mexicana* (blue). See Materials and Methods and Table S6 for the GenBank accession numbers of the sequences used. **B.** Average intrinsic curvature of the five core promoters of rRNA genes shown in A. At each nucleotide position, dots and vertical bars represent average curvature and standard deviation, respectively. The vertical dashed lines at +1 in both panels mark the position of the TSP. Curvature is expressed as degrees (deg.) per helical turn. TSP, transcription start point.

The shifted intrinsic curvature profile observed in the *L. major* promoter shown in Figure 1A prompted us to study whether this was a peculiarity of this species. Since the core promoter and TSP for rRNA genes in *Leishmania* other than *L. major* have been also experimentally determined (*e.g.*, *L. donovani* [19], *L. amazonensis*, *L. mexicana* [20], and *L. chagasi* [21]), we investigated the curvature profiles in these species as well. These sequences have a nucleotide identity ranging 0.5–1 (Table S1). Despite the varied sequence similarity, they showed a very similar intrinsic curvature profile, which consists of two peaks enclosing a valley with a minimum located 10–14 nt upstream to the TSP (Figure 2). Therefore, such a pattern could be considered as a genus-specific characteristic of the rRNA core promoter, which distinguishes *Leishmania* from *T. cruzi* and *T. brucei*.

### The rRNA gene promoter curvature is conserved within *T. cruzi* strains

**Figure 3:**
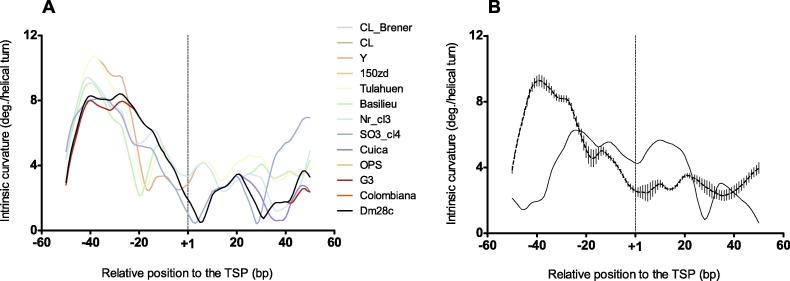
**Intrinsic curvature profiles around the TSPs for rRNA promoter genes in *T. cruzi*** Intrinsic curvature for the core promoters of indicated rRNA genes in the 100-bp regions centered at the TSP in *T. cruzi*. **A.** Individual intrinsic curvature for the core promoters of rRNA genes of 13 different *T. cruzi* strains. In cases where sequences are identical in the analyzed region, only one profile was plotted (see Table S2). See Materials and Methods and Table S6 for the GenBank accession numbers of the sequences used. **B.** Average intrinsic curvature of the core promoters of the rRNA genes shown in A, besides the pattern of a non-active rRNA core promoter described in [25] is shown as a black solid line. Dots and vertical bars at each nucleotide position represent average curvature and standard deviation, respectively. The vertical dashed lines at +1 in A and B mark the position of the TSP. Curvature is expressed as degrees (deg.) per helical turn. TSP, transcription start point.

In the case of *T. cruzi*, the rRNA promoter for various strains has been sequenced [22] and their TSPs have been experimentally determined [23], [24]. The nucleotide identity among these sequences ranges 0.75–1 (Table S2). We found that the intrinsic curvature pattern of the core promoters is conserved among these various *T. cruzi* strains (Figure 3**A**). Particularly, a peak or shoulder of intrinsic curvature is observed at position −10 of the different strains and is conspicuous in the consensus (Figure 3**B**). As a proof of principle, we compared the curvature of an early proposed TSP of the *T. cruzi* Cl strain [25], located approximately 300 nucleotides downstream from the currently accepted TSP, which was demonstrated to contribute little, if any, to the transcriptional activity [24], [26]. Accordingly, we found that the intrinsic curvature pattern for this controversial promoter is quite divergent from the consensus (see the solid line in Figure 3B).

**Supplementary Figure S2:**
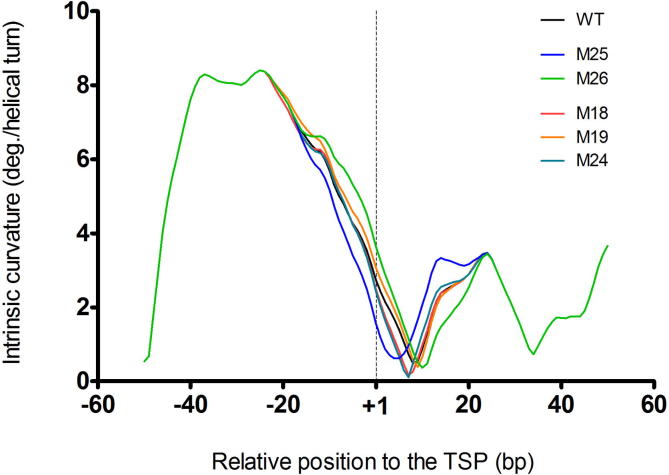
**Intrinsic curvature profiles around the TSPs for rRNA gene promoter mutants in *T. cruzi*** Intrinsic curvature for the mutant core promoters of indicated rRNA genes in the 100-bp regions centered at the TSP. See Materials and Methods and Table S6 for the GenBank accession numbers of the sequences used. The vertical dashed line at +1 marks the position of the TSP. Curvature is expressed as degrees (deg.) per helical turn. TSP, transcription start point.

**Figure 4:**
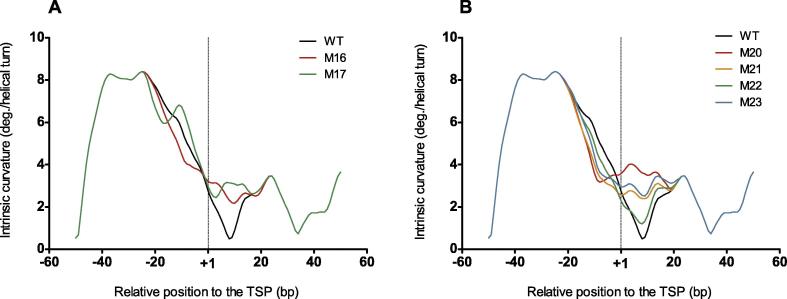
**Intrinsic curvature profiles around the TSPs for mutant rRNA genes promoters in *T. cruzi*** Intrinsic curvature for the core promoters of indicated rRNA genes in the 100-bp regions centered at the TSP in *T. cruzi*. **A.** Mutations at the transcription start region (see [27]). M16 (Construct 16) is a down-regulating mutant, whereas M17 (Construct 17) is an up-regulating mutant [27]. **B.** Four down-regulating point mutations at the transcription start region, M20–M23 (see [27]). The vertical dashed lines at +1 in both panels mark the position of the TSP. Curvature is expressed as degrees (deg.) per helical turn. TSP, transcription start point.

Given the availability of studies on mutations near the TSP of the rRNA gene from the Mexican strain la Cruz Jalisco (*T. cruzi* I) [27], we sought to correlate effect of these mutations on the transcriptional activity with their ability to modify the intrinsic curvature pattern of the core promoter. Using a reporter gene approach, Figueroa-Angulo et al. [27] found that the simultaneous substitution of six base pairs (bp) around the TSP (−1 to +5) produced a virtually inactive promoter (Construct 16). Interestingly, the intrinsic curvature pattern of this construct́s core promoter clearly differs from the wild type (Figure 4**A**), with obvious loss of intrinsic curvature at the position −10. Meanwhile, the point mutation at position −3 (Construct 17), which increases the promoter activity (124%), shows an increased intrinsic curvature at this position (Figure 4A). Figueroa-Angulo et al. also observed that substitutions at the three experimentally-detected TSPs (+1, +2, and +4, Constructs 20, 21, and 23) produced defective promoters (remaining activity of 10%, 41%, and 28%, respectively). Besides, the substitution at the position +3 (Constructs 22) also produced a reduced promoter activity (remaining activity of 38%) [27]. We found that these four substitutions (Constructs 20–23) yield a decreased intrinsic curvature at the position −10 (Figure 4**B**), reinforcing the relevance of the intrinsic curvature at this position. In addition, these mutations cause a reduction of the deepness of the intrinsic curvature valley observed between positions +1 and +15 in comparison with the wild type sequence. Concordantly, two nucleotide substitutions that do not markedly modify the wild type intrinsic curvature consensus (Constructs 25 and 26) only slightly affect the promoter activity (87% and 83%, respectively) (Figure S2). The remaining three substitutions tested in this study (Constructs 18, 19, and 24) reduced the promoter activity without obvious alteration of the curvature pattern relative to the wild type sequence (Figure S2), suggesting that additional perturbations of the promoter, such as loss of primary sequence motives, might take place for these mutants. In summary, the transcriptional activities for 8 out of the 11 mutants studied are in agreement with their intrinsic curvature profiles.

### The promoters of most RNAPI transcribed protein-coding genes RNAPI in *T. brucei* share the conserved eukaryotic curvature

**Figure 5:**
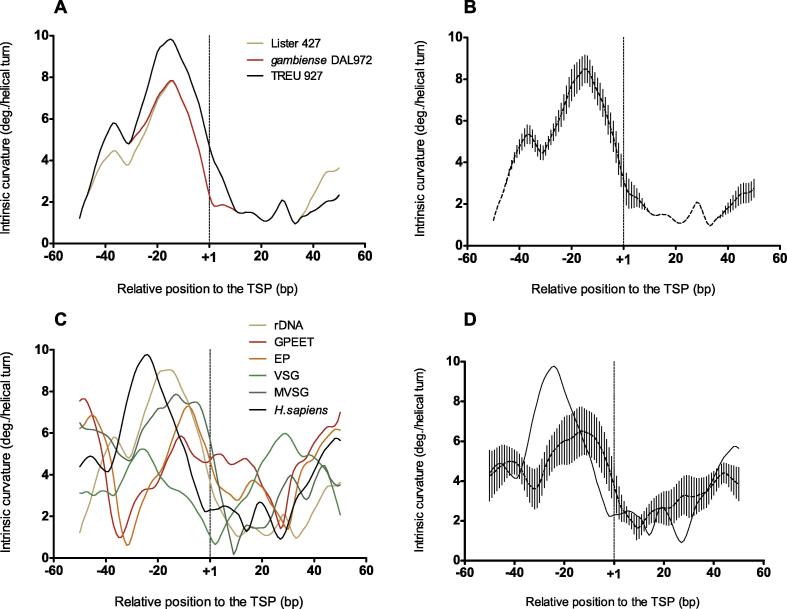
**Intrinsic curvature profiles around the TSPs for genes transcribed by RNAPI in *T. brucei*** Intrinsic curvature for the core promoters of indicated rRNA genes in the 100-bp regions centered at the TSP in *T. brucei*. **A.** Intrinsic curvature of the individual core promoters of rRNA genes for *T. brucei* Lister 427, *T. brucei* TREU 927, *T. brucei* gambiense DAL972. **B.** Average intrinsic curvature of the core promoters of rRNA genes shown in A. Dots and vertical bars at each nucleotide position represent average curvature and standard deviation, respectively. **C.** Individual intrinsic curvature around the TSP for all the RNAPI-transcribed promoters in *T. brucei*. Sequence FM162566 (green) was chosen as a representative *VSG* BES promoter. See Materials and Methods and Table S6 for the GenBank accession numbers of the remaining sequences. **D.** Average intrinsic curvature average around the TSP for all the genes transcribed by RNAPI in *T. brucei* except *GPEET* (rDNA, *VSG*, *MVSG*, and *EP1*), vertical lines represent the standard deviation for each position. In panels C and D, the intrinsic curvature of human rRNA promoter was included as a reference (solid black line). The vertical dashed lines at +1 in panels A−D mark the position of the TSP. Curvature is expressed as degrees (deg.) per helical turn. TSP, transcription start point.

As for *T. cruzi*, analysis of *T. brucei* genomic data (*T. brucei* Lister 427, *T. brucei* TREU 927, and *T. brucei gambiense* DAL972) showed that rRNA core promoters have highly similar sequence (identity 0.97–0.99, Table S3) and curvature (Figure 5**A** and **B**). In order to examine whether this curvature profile is also conserved at the promoters of the protein-coding genes that are transcribed by RNAPI*,* we analyzed the intrinsic curvature of the core promoters of the genes encoding major surface proteins: the VSGs of the metacyclic and bloodstream form of the parasite, and the acidic repetitive proteins GPEET and EP1 of the procyclic form. We found that the core promoters of the genes encoding the metacyclic and bloodstream VSGs, and the *EP1*, share a curvature pattern that is closely similar to that of the rRNA promoters (Figure 5**C** and **D**), in spite of the lack of nucleotide sequence conservation [28] (identity 0.31–0.47, Table S4). Meanwhile, the curvature displayed by the *GPEET* promoter is different, which lacks the valley of curvature toward the TSP as observed in the other promoters (Figure 5C and D, dotted line). This difference is noticeable, despite its high sequence similarity to the *EP1* promoter (identity of 0.945, Table S4).

**Supplementary Figure S3:**
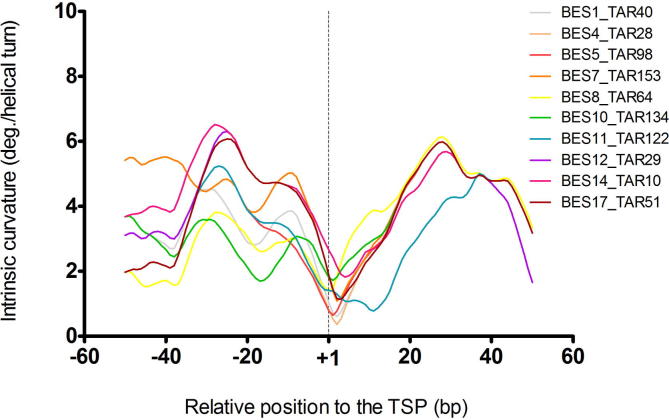
**Intrinsic curvature profiles around the TSPs for *VSG* genes in *T. brucei*** Individual intrinsic curvature around the TSP for the 24 BES promoters described in [15] for *T. brucei* strain 427. The 100-bp region shown is centered at the TSP indicated as +1. In cases where sequences are identical in the analyzed region, only one profile was plotted (see Table S5). See Materials and Methods and Table S6 for the GenBank accession numbers of the sequences used. The vertical dashed line at +1 marks the position of the TSP. Curvature is expressed as degrees (deg.) per helical turn. TSP, transcription start point.

Reinforcing these findings, all the core promoters of the *VSG* BES from the large repertoire identified in *T. brucei* 427 [15] display the conserved rRNA curvature profile, although sequence variations are present (Figure S3 and Table S5).

## Discussion

Trypanosomatids are interesting biological models for basic research due to their remarkable deviations from eukaryotic molecular paradigms. Transcription initiation constitutes one of such peculiarities, where a group of highly expressed *T. brucei* protein-coding genes (*VSG* and *procyclins*) is known to be transcribed by RNAPI. The trypanosomatid RNAPI promoters of the rRNA and of the *T. brucei* RNAPI dependent protein-coding genes have been defined and well characterized (recently reviewed in [9]). It is well established that signals directing transcription initiation by RNAPI are constituted by evolutionarily-conserved DNA secondary structures rather than the sequences [10], [11], [12]. Particularly, existence of peaks of intrinsic curvature at the proximity of the TSP has been described in several species [10], [11], [12]. Nevertheless, the intrinsic curvature of RNAPI core promoter has not yet been studied in trypanosomatids. This is unfortunate since the conservation of a consensus pattern of promoter curvature in this early phylogenetic branch may provide further support to the secondary structure signaling of eukaryotic RNAPI core promoter. Furthermore, *T. brucei* is a unique organism, whose RNAPI is proved to drive the transcription of protein-coding genes [9]. Therefore, in order to further the current understanding of the molecular mechanisms involved in transcription initiation by RNAPI, we analyzed the intrinsic curvature of the Tritryp rRNA gene core promoters.

Our findings agree with the previous hypothesis that intrinsic curvature is fundamental for rRNA core promoter function [11], and extend the conserved conformational pattern to the ancient eukaryotic protozoans. Indeed, a common profile of high peaks of intrinsic curvature surrounding a valley of reduced curvature close to the TSP was found for *T. cruzi*, *T. brucei*, and *L. major*. In addition, the Tritryp pattern closely resembles that of the human counterpart. Although the predicted curvature of the *Leishmania* promoter is relatively dissimilar, which is comparatively shifted 10–14 nt upstream, it is well conserved among *L. donovani*, *L. amazonensis*, *L. mexicana*, *L. chagasi*, and *L. major*. This observation is in agreement with lack of heterologous RNAPI promoter activity between *Leishmania* and *Trypanosomes* [29], which contrasts with the exchangeability within *Leishmania* genus and within *Trypanosome* promoters. Indeed, for these cases, strain heterologous RNAPI promoters have been proved to be functional [21]. It is worth mentioning that the *Leishmania* genome exhibits structural peculiarities that set these organisms apart from other eukaryotes and even from their close related trypanosomatids. *Leishmania* has different base composition, unique association of G + C skew with the coding strand [30], and the particular genomic curvature showing a low proportion of high intrinsic curvature regions [5], which may underlay this observation for reasons still not understood. According to the current study, we speculate that the existence of a certain level of species-specific RNAPI promoter curvature might be related to the particular genome characteristics.

The pattern of high peaks surrounding a valley of intrinsic curvature close to the TSP at the core promoter of the rRNA gene is also conserved within at least 10 different *T. cruzi* strains. Nonetheless, the relevance of the conformational pattern is not only supported by its wide conservation among parasite strains, but also by its functional correlation with the promoter activity of the previously-reported mutants [27]. Remarkably, a substitution, which increases the *in vitro* promoter activity (Construct 17 [27]), is present in several *T. cruzi* strains (Y, 150zd, Tulahuen, Basilieu, Nr_cl3, and SO3_cl4) and leads to the increased intrinsic curvature at position −10. On the other hand, five downregulating mutations (Construct 16, 20, 21, 22, and 23 [27]) reduce the expected intrinsic curvature peak/shoulder at position −10 and smoothen the deepness of the valley close to the TSP. These changes may set flexibility constraints at the initiation regions, which in turn may prevent the bending that precedes transcription initiation in RNAPI promoters, thus contributing to the loss of promoter activity. Meanwhile, the intrinsic curvature fails to explain the reduction in transcription activity for three point mutations, which apparently do not introduce changes at this level (Figure S2). It is worth to note that mutations may also affect other steps of the transcription machinery assembly and functioning. Remarkably, none of the mutants that are predicted to modify DNA curvature at the core promoter were neutral to the transcriptional activity assay. Globally, these findings point out to the putative usefulness of the intrinsic curvature analysis for the prediction of functional RNAPI promoters.

The genes encoding the abundant stage-specific glycoproteins exposed at the cell surface (VSGs and EP/GPEET procyclins) are also transcribed by RNAPI in *T. brucei* [9]. Interestingly, the core promoters of these genes exhibit a curvature profile similar to the eukaryotic rRNA gene core promoter consensus. This indicates that the secondary structure of the RNAPI-driven promoters is an inherent requirement for transcription machinery assembly independent of the nature of the transcribed product (either rRNA or mRNA).

Early studies have shown that, in spite of the absence of sequence conservation, functional RNAPI hybrid promoters could be obtained in trypanosomatids depending on nucleotide spacing constraints [16]. These results could be explained if promoter functionality relies on DNA conformational requirements such as the curvature features revealed by our analysis. While the requisite of specific TFs for the assembly of RNAPI machinery at the different core promoters could justify the absence of sequence conservation, competition experiments have evidenced that this is not the case, because TFs seem to be greatly shared among these promoters [16]. Since different nucleotide sequence arrangements can lead to similar secondary structures [31], our observations are compatible with the existence of conserved conformational signals at the RNAPI promoter. The absence of primary sequence elements observed in rRNA promoters of eukaryotes is remarked in *T. brucei*, where the promoters for rRNA and protein-coding genes have extensively diverged in sequence composition. However, our results suggest that a selection pressure acts to strictly maintain their conformation. Indeed, different variants with no consequences on the intrinsic curvature are naturally present in the *T. brucei VSG* core promoters. These constitute “conformationally-silent single nucleotide variants”. Meanwhile, artificially-induced single point mutations affecting the intrinsic curvature pattern of the *T. cruzi* rRNA gene promoter have been proved to disturb the transcriptional activity.

While intrinsic curved DNA most likely functions as a signal for TF recognition or interaction and/or facilitates the formation of the open promoter complex [31], the exact role of such curvature in trypanosomatid RNAPI core promoters needs to be further addressed.

In summary, our results indicate that the RNAPI core promoters of kinetoplastids conserve the eukaryotic intrinsic curvature features, not only for the rRNA genes, but also for the protein-coding genes transcribed by RNAPI.

## Materials and methods

Promoter sequences were aligned and the similarities were calculated by using the Emboss suite [32]. To calculate the sequence-dependent DNA curvature, we used “bend-it®”, an automated prediction resource based on algorithms developed using the helical asymmetry of trinucleotides, available at DNAtools server [33] (http://www.icgeb.trieste.it/dna). The curvature is expressed as degrees per helical turn (10.5°/helical turn = 1°/basepair).

A summary of the accession numbers of the sequences used in the present study is given in Table S6.

We obtained the rRNA promoter region for *T. brucei* Lister strain 427 from the NCBI (GenBank accession No. AF416290.1), whose TSP was previously mapped [34]. The corresponding regions for *T. brucei* TREU927 and *T. brucei* gambiense DAL972 were obtained by blast homology search in the TritrypDB database (TSPs are located in chromosome 3, base 901,732 and chromosome 7, base 1,995,894, respectively).

Promoter sequences described for *T. cruzi* strains CL Brener, CL, Y, 150zd, Tulahuen, Basilieu, Nr cl3, SO3 cl4, Cuica, OPS, G3, Colombiana, and Dm28c [22] were analyzed. Their sequences have the consecutive GenBank accession No. (U89776.1–U89788.1). The major TSP for the *T. cruzi* promoter mapped by Figueroa-Angulo et al. was used [24], [26].

*Leishmania* promoter sequences used in this study were retrieved from the NCBI database as well (GenBank accession No. AF421555 for *L. major* [20]*,* AF421554 for *L. mexicana* [20], L38572.1 for *L. donovani* [35], U21687 for *L amazonensis* [36], and U42465 for *L. donovani chagasi* [21]). The TSPs were defined according to Andrade-Stempliuk et al. [20].

For the analysis of the *T. brucei* surface protein genes promoters, we obtained the sequences of all the BES of *T. brucei* 427 described by Hertz-Fowler et al. [15], which are available through the EMBL-EBI (accession No. FM162566–FM162583). The TSPs were mapped according to Zomerdijk et al. [37]. In addition, promoter sequences for genes encoding metacyclic VSGs were obtained from the NCBI (GenBank accession No*.* AJ486955.1) for the analysis with TSP experimentally determined previously [38]. Similarly, for genes encoding procyclins, *GPEET* (GenBank accession No. S60066.1) and *EP1* (GenBank accession No. M38222.1) were analyzed [16].

The previously-characterized human rRNA promoter (GenBank accession No. X01547 [39]) was used as a reference [11], whereas 35 randomly-selected human RNAPII promoters were downloaded from the Eukaryotic Promoter Database (http://epd.vital-it.ch/) as nonspecific controls (gene IDs: ABI3BP_1, ACKR3_1, ACTB_1, ACTC_1, ADM_1, AGR2_1, AKR1B1_1, DEPP_1, ELF3_1, EMR2_2, FGA_1, FLT1_1, FOS_1, GAL_1, H2AFX_1, H32_1, HSPCA_1, ID1_1, IFNA2_1, IGF2_7, IGFBP3_1, LTB_1, RCAN1_3, RNASE2_2, RPS18_1, S100A12_1, SDC4_1, TLR6_1, TM4SF20_1, TMED1_1, TNFRSF10D_1, UBD_1, WFDC2_1, WISP2_1, and YBX3_1).

## Authors’ contributions

BG conceived and designed the study. PS obtained the data, whereas PS, MD, and BG analyzed and/or interpreted the data. All authors participated in manuscript writing, read and approved the final manuscript.

## Competing interests

The authors have declared no competing interests.

## Figures and Tables

**Table 1:** Nucleotide similarity matrix for the TriTryp promoter sequences

	***H. sapiens***	***L. major***	***T. brucei***	***T. cruzi***
*H. sapiens*	1			
*L. major*	0.29	1		
*T. brucei*	0.30	0.27	1	
*T. cruzi*	0.41	0.34	0.33	1

*Note: L. major* (AF421555.1); *T. cruzi* (U89782.1); *T. brucei* (AF416290.1). *H. sapiens* (X01547) was used as an external control.

## Supplementary material

Supplementary Table S1 Nucleotide similarity matrix for the *Leishmania* rRNA promoters analyzed.

Supplementary Table S2 Nucleotide similarity matrix for the *T. cruzi* rRNA promoters analyzed.

Supplementary Table S3 Nucleotide similarity matrix for the *T. brucei* rRNA promoters analyzed.

Supplementary Table S4 Nucleotide similarity matrix for the *T. brucei* RNAPI promoters analyzed.

Supplementary Table S5 Nucleotide similarity matrix for the *T. brucei* BES promoters analyzed.

Supplementary Table S6 Accession numbers for sequences used in the study.
