# Supplementary material for: Conserved Curvature of RNA Polymerase I Core Promoter Beyond rRNA Genes: The Case of the Tritryps
Source: Genomics Proteomics Bioinformatics. 2015 Dec 21;13(6):355–63. doi: 10.1016/j.gpb.2015.09.005 (PMC4747651; doi:10.1016/j.gpb.2015.09.005)
Supplement: Supplementary Table S1 — Nucleotide similarity matrix for the Leishmania rRNA promoters analyzed. [file mmc1.docx]

**Table S1 Nucleotide similarity matrix for the Leishmania rRNA promoters analyzed**

|  | ***L. major*** | ***L. amazonensis*** | ***L. mexicana*** | ***L. donovani*** | ***L. donovani chagasi*** |
| --- | --- | --- | --- | --- | --- |
| ***L. major*** | 1 |  |  |  |  |
| ***L. amazonensis*** | 0.99 | 1 |  |  |  |
| ***L. mexicana*** | 0.882 | 0.892 | 1 |  |  |
| ***L. donovani*** | 0.557 | 0.557 | 0.539 | 1 |  |
| ***L. donovani chagasi*** | 0.557 | 0.557 | 0.539 | 1 | 1 |

*Note:* See Table S6 for sequence IDs.
