# Supplementary material for: Conserved Curvature of RNA Polymerase I Core Promoter Beyond rRNA Genes: The Case of the Tritryps
Source: Genomics Proteomics Bioinformatics. 2015 Dec 21;13(6):355–63. doi: 10.1016/j.gpb.2015.09.005 (PMC4747651; doi:10.1016/j.gpb.2015.09.005)
Supplement: Supplementary Table S2 — Nucleotide similarity matrix for the T. cruzi rRNA promoters analyzed. [file mmc2.docx]

**Table S2 Nucleotide similarity matrix for the *T. cruzi* rRNA promoters analyzed**

|  | **Y** | **Tulahuen** | **SO3_cl4** | **OPS** | **Nr_cl3** | **G3** | **Dm28c** | **Cuica** | **Colombiana** | **CL_Brener** | **CL** | **Basilieu** | **150zd** |
| --- | --- | --- | --- | --- | --- | --- | --- | --- | --- | --- | --- | --- | --- |
| **Y** | 1 |  |  |  |  |  |  |  |  |  |  |  |  |
| **Tulahuen** | 0.96 | 1 |  |  |  |  |  |  |  |  |  |  |  |
| **SO3_cl4** | 0.805 | 0.823 | 1 |  |  |  |  |  |  |  |  |  |  |
| **OPS** | 0.759 | 0.776 | 0.871 | 1 |  |  |  |  |  |  |  |  |  |
| **Nr_cl3** | 0.97 | 0.95 | 0.805 | 0.759 | 1 |  |  |  |  |  |  |  |  |
| **G3** | 0.769 | 0.786 | 0.861 | 0.99 | 0.769 | 1 |  |  |  |  |  |  |  |
| **Dm28c** | 0.759 | 0.776 | 0.871 | 1 | 0.759 | 0.99 | 1 |  |  |  |  |  |  |
| **Cuica** | 0.75 | 0.766 | 0.861 | 0.99 | 0.75 | 0.98 | 0.99 | 1 |  |  |  |  |  |
| **Colombiana** | 0.759 | 0.776 | 0.871 | 1 | 0.759 | 0.99 | 1 | 0.99 | 1 |  |  |  |  |
| **CL_Brener** | 0.96 | 1 | 0.823 | 0.776 | 0.95 | 0.786 | 0.776 | 0.766 | 0.776 | 1 |  |  |  |
| **CL** | 0.96 | 1 | 0.823 | 0.776 | 0.95 | 0.786 | 0.776 | 0.766 | 0.776 | 1 | 1 |  |  |
| **Basilieu** | 0.97 | 0.97 | 0.815 | 0.769 | 0.98 | 0.778 | 0.769 | 0.759 | 0.769 | 0.97 | 0.97 | 1 |  |
| **150zd** | 0.98 | 0.98 | 0.815 | 0.769 | 0.97 | 0.778 | 0.769 | 0.759 | 0.769 | 0.98 | 0.98 | 0.99 | 1 |

*Note:* See Table S6 for sequence IDs.
