# Supplementary material for: Conserved Curvature of RNA Polymerase I Core Promoter Beyond rRNA Genes: The Case of the Tritryps
Source: Genomics Proteomics Bioinformatics. 2015 Dec 21;13(6):355–63. doi: 10.1016/j.gpb.2015.09.005 (PMC4747651; doi:10.1016/j.gpb.2015.09.005)
Supplement: Supplementary Table S3 — Nucleotide similarity matrix for the T. brucei rRNA promoters analyzed. [file mmc3.docx]

**Table S3 Nucleotide similarity matrix for the *T. brucei* rRNA promoters analyzed**

|  | ***T. brucei* Lister 427** | ***T. brucei* TREU 927** | ***T. brucei* gambiense DAL972** |
| --- | --- | --- | --- |
| ***T. brucei* Lister 427** | 1 |  |  |
| ***T. brucei* TREU 927** | 0.97 | 1 |  |
| ***T. brucei* gambiense DAL972** | 0.99 | 0.98 | 1 |

*Note:* See Table S6 for sequence IDs.
