# Supplementary material for: Conserved Curvature of RNA Polymerase I Core Promoter Beyond rRNA Genes: The Case of the Tritryps
Source: Genomics Proteomics Bioinformatics. 2015 Dec 21;13(6):355–63. doi: 10.1016/j.gpb.2015.09.005 (PMC4747651; doi:10.1016/j.gpb.2015.09.005)
Supplement: Supplementary Table S4 — Nucleotide similarity matrix for the T. brucei RNAPI promoters analyzed. [file mmc4.docx]

**Table S4 Nucleotide similarity matrix for the *T. brucei* RNAPI promoters analyzed**

|  | **GPEET** | **EP1** | **rDNA** | **VSG** | **MVSG** |
| --- | --- | --- | --- | --- | --- |
| **GPEET** | 1 |  |  |  |  |
| **EP1** | 0.945 | 1 |  |  |  |
| **rDNA** | 0.474 | 0.453 | 1 |  |  |
| **VSG** | 0.37 | 0.34 | 0.38 | 1 |  |
| **MVSG** | 0.309 | 0.309 | 0.371 | 0.37 | 1 |

*Note:* See Table S6 for sequence IDs.
