# Supplementary material for: Conserved Curvature of RNA Polymerase I Core Promoter Beyond rRNA Genes: The Case of the Tritryps
Source: Genomics Proteomics Bioinformatics. 2015 Dec 21;13(6):355–63. doi: 10.1016/j.gpb.2015.09.005 (PMC4747651; doi:10.1016/j.gpb.2015.09.005)
Supplement: Supplementary Table S5 — Nucleotide similarity matrix for the T. brucei BES promoters analyzed. [file mmc5.docx]

**Table S5 Nucleotide similarity matrix for the *T. brucei* BES promoters analyzed**

|  | **1_TAR40** | **2_TAR129** | **3_TAR15_a** | **3_TAR15_b** | **4_TAR28** | **5_TAR98** | **7_TAR153_a** | **7_TAR153_b** | **7_TAR65** | **8_TAR64** | **10_TAR134_a** | **10_TAR134_b** | **11_TAR122** | **12_TAR29_a** | **12_TAR29_b** | **13_TAR56_a** | **13_TAR56_b** | **14_TAR10** | **15_TAR126_a** | **15_TAR126_b** | **17_TAR51_a** | **17_TAR51_b** | **17_TAR59_a** | **17_TAR59_b** |
| --- | --- | --- | --- | --- | --- | --- | --- | --- | --- | --- | --- | --- | --- | --- | --- | --- | --- | --- | --- | --- | --- | --- | --- | --- |
| **1_TAR40** | 1 |  |  |  |  |  |  |  |  |  |  |  |  |  |  |  |  |  |  |  |  |  |  |  |
| **2_TAR129** | 1 | 1 |  |  |  |  |  |  |  |  |  |  |  |  |  |  |  |  |  |  |  |  |  |  |
| **3_TAR15_a** | 1 | 1 | 1 |  |  |  |  |  |  |  |  |  |  |  |  |  |  |  |  |  |  |  |  |  |
| **3_TAR15_b** | 1 | 1 | 1 | 1 |  |  |  |  |  |  |  |  |  |  |  |  |  |  |  |  |  |  |  |  |
| **4_TAR28** | 0.95 | 0.95 | 0.95 | 0.95 | 1 |  |  |  |  |  |  |  |  |  |  |  |  |  |  |  |  |  |  |  |
| **5_TAR98** | 0.94 | 0.94 | 0.94 | 0.94 | 0.99 | 1 |  |  |  |  |  |  |  |  |  |  |  |  |  |  |  |  |  |  |
| **7_TAR153_a** | 0.96 | 0.96 | 0.96 | 0.96 | 0.93 | 0.92 | 1 |  |  |  |  |  |  |  |  |  |  |  |  |  |  |  |  |  |
| **7_TAR153_b** | 0.96 | 0.96 | 0.96 | 0.96 | 0.93 | 0.92 | 1 | 1 |  |  |  |  |  |  |  |  |  |  |  |  |  |  |  |  |
| **7_TAR65** | 1 | 1 | 1 | 1 | 0.95 | 0.94 | 0.96 | 0.96 | 1 |  |  |  |  |  |  |  |  |  |  |  |  |  |  |  |
| **8_TAR64** | 0.94 | 0.94 | 0.94 | 0.94 | 0.93 | 0.92 | 0.92 | 0.92 | 0.94 | 1 |  |  |  |  |  |  |  |  |  |  |  |  |  |  |
| **10_TAR134_a** | 0.97 | 0.97 | 0.97 | 0.97 | 0.96 | 0.95 | 0.93 | 0.93 | 0.97 | 0.93 | 1 |  |  |  |  |  |  |  |  |  |  |  |  |  |
| **10_TAR134_b** | 0.98 | 0.98 | 0.98 | 0.98 | 0.95 | 0.94 | 0.94 | 0.94 | 0.98 | 0.94 | 0.99 | 1 |  |  |  |  |  |  |  |  |  |  |  |  |
| **11_TAR122** | 0.93 | 0.93 | 0.93 | 0.93 | 0.98 | 0.97 | 0.93 | 0.93 | 0.93 | 0.93 | 0.94 | 0.93 | 1 |  |  |  |  |  |  |  |  |  |  |  |
| **12_TAR29_a** | 1 | 1 | 1 | 1 | 0.95 | 0.94 | 0.96 | 0.96 | 1 | 0.94 | 0.97 | 0.98 | 0.93 | 1 |  |  |  |  |  |  |  |  |  |  |
| **12_TAR29_b** | 0.95 | 0.95 | 0.95 | 0.95 | 0.98 | 0.97 | 0.95 | 0.95 | 0.95 | 0.91 | 0.96 | 0.95 | 0.96 | 0.95 | 1 |  |  |  |  |  |  |  |  |  |
| **13_TAR56_a** | 1 | 1 | 1 | 1 | 0.95 | 0.94 | 0.96 | 0.96 | 1 | 0.94 | 0.97 | 0.98 | 0.93 | 1 | 0.95 | 1 |  |  |  |  |  |  |  |  |
| **13_TAR56_b** | 0.95 | 0.95 | 0.95 | 0.95 | 0.98 | 0.97 | 0.95 | 0.95 | 0.95 | 0.91 | 0.96 | 0.95 | 0.96 | 0.95 | 1 | 0.95 | 1 |  |  |  |  |  |  |  |
| **14_TAR10** | 0.96 | 0.96 | 0.96 | 0.96 | 0.95 | 0.94 | 0.94 | 0.94 | 0.96 | 0.9 | 0.97 | 0.96 | 0.93 | 0.96 | 0.97 | 0.96 | 0.97 | 1 |  |  |  |  |  |  |
| **15_TAR126_a** | 0.95 | 0.95 | 0.95 | 0.95 | 0.98 | 0.97 | 0.95 | 0.95 | 0.95 | 0.91 | 0.96 | 0.95 | 0.96 | 0.95 | 1 | 0.95 | 1 | 0.97 | 1 |  |  |  |  |  |
| **15_TAR126_b** | 0.95 | 0.95 | 0.95 | 0.95 | 0.98 | 0.97 | 0.95 | 0.95 | 0.95 | 0.91 | 0.96 | 0.95 | 0.96 | 0.95 | 1 | 0.95 | 1 | 0.97 | 1 | 1 |  |  |  |  |
| **17_TAR51_a** | 1 | 1 | 1 | 1 | 0.95 | 0.94 | 0.96 | 0.96 | 1 | 0.94 | 0.97 | 0.98 | 0.93 | 1 | 0.95 | 1 | 0.95 | 0.96 | 0.95 | 0.95 | 1 |  |  |  |
| **17_TAR51_b** | 0.94 | 0.94 | 0.94 | 0.94 | 0.95 | 0.94 | 0.92 | 0.92 | 0.94 | 0.94 | 0.95 | 0.94 | 0.93 | 0.94 | 0.97 | 0.94 | 0.97 | 0.96 | 0.97 | 0.97 | 0.94 | 1 |  |  |
| **17_TAR59_a** | 1 | 1 | 1 | 1 | 0.95 | 0.94 | 0.96 | 0.96 | 1 | 0.94 | 0.97 | 0.98 | 0.93 | 1 | 0.95 | 1 | 0.95 | 0.96 | 0.95 | 0.95 | 1 | 0.94 | 1 |  |
| **17_TAR59_b** | 0.94 | 0.94 | 0.94 | 0.94 | 0.95 | 0.94 | 0.92 | 0.92 | 0.94 | 0.94 | 0.95 | 0.94 | 0.93 | 0.94 | 0.97 | 0.94 | 0.97 | 0.96 | 0.97 | 0.97 | 0.94 | 1 | 0.94 | 1 |

*Note:* See Table S6 for sequence IDs.
