# Supplementary material for: Conserved Curvature of RNA Polymerase I Core Promoter Beyond rRNA Genes: The Case of the Tritryps
Source: Genomics Proteomics Bioinformatics. 2015 Dec 21;13(6):355–63. doi: 10.1016/j.gpb.2015.09.005 (PMC4747651; doi:10.1016/j.gpb.2015.09.005)
Supplement: Supplementary Table S6 — Accession numbers for sequences used in the study. [file mmc6.docx]

**Table S6 Accession numbers for sequences used in the study**

| **Accession No.** | **Description** | **Organism** | **Strain** |
| --- | --- | --- | --- |
| U89776.1 | rDNA promoter | *T. cruzi* | CL Brener |
| U89777.1 | rDNA promoter | *T. cruzi* | CL |
| U89778.1 | rDNA promoter | *T. cruzi* | Y |
| U89779.1 | rDNA promoter | *T. cruzi* | 150zd |
| U89780.1 | rDNA promoter | *T. cruzi* | Tulahuen |
| U89781.1 | rDNA promoter | *T. cruzi* | Basilieu |
| U89782.1 | rDNA promoter | *T. cruzi* | Nr cl3 |
| U89783.1 | rDNA promoter | *T. cruzi* | SO3 cl4 |
| U89784.1 | rDNA promoter | *T. cruzi* | Cuica |
| U89785.1 | rDNA promoter | *T. cruzi* | OPS |
| U89786.1 | rDNA promoter | *T. cruzi* | G3 |
| U89787.1 | rDNA promoter | *T. cruzi* | Colombiana |
| U89788.1 | rDNA promoter | *T. cruzi* | Dm28c |
| AF421555 | rDNA promoter | *L. major* |  |
| AF421554 | rDNA promoter | *L. mexicana* |  |
| L38572.1 | rDNA promoter | *L. donovani* |  |
| U21687 | rDNA promoter | *L. amazonensis* |  |
| U42465 | rDNA promoter | *L. donovani chagasi* |  |
| AF416290.1 | rDNA promoter | *T. brucei* | Lister 427 |
| FM162566−FM162583 | BES promoters | *T. brucei* | Lister 427 |
| AJ486955.1 | Metacyclic VSG promoter | *T. brucei* | EATRO 795 |
| S60066.1 | GPEET promoter | *T. brucei* | Lister 427 |
| M38222.1 | EP1 promoter | *T. brucei* | 118 |
| X01547 | rDNA promoter | *H. sapiens* |  |
